# Uterine factors modify the association between embryo transfer depth and clinical pregnancy

**DOI:** 10.1038/s41598-022-18636-4

**Published:** 2022-08-22

**Authors:** Xiaohua Sun, Jiali Cai, Lanlan Liu, Haixiao Chen, Xiaoming Jiang, Jianzhi Ren

**Affiliations:** The Affiliated Chenggong Hospital of Xiamen University, Xiamen, 361002 Fujian China

**Keywords:** Reproductive disorders, Endocrine reproductive disorders

## Abstract

The embryo transfer depth may affect the chance of pregnancy. However, embryo dislodging caused by uterine contraction may occur after the transfer. The aim of the retrospective study was to investigate whether the factors associated with uterine contractilities, such as endometrial thickness and progesterone elevation, affect the association between transfer depth and implantation. A total of 7849 fresh transfer cycles on conventional stimulation in a single in vitro fertilization (IVF) center during the period 2013–2015 was reviewed. Patients were categorized according to quartiles of embryo transfer depth (≤ 9 mm, n = 1735, 9.1–11 mm, n = 2557, 11.1–14 mm, n = 1933, ≥ 1.4 mm, n = 1624, respectively). Adjusted for confounding factors, the adjusted odds ratio (aOR) (95% confidence interval, CI) for clinical pregnancy was 0.90 (0.79–1.02), 0.86 (0.74–0.99), and 0.70 (0.60–0.82) respectively in quartiles 2 through 4, comparing with quartile 1. However, the aORs were significantly increased when the endometrial thickness was < 8 mm. In comparison with that in the cycles with a normal endometrial thickness (8–11 mm), the aORs comparing quartiles 2 through 4 with quartile 1 in the cycles with an endometrial thickness < 8 mm increased from 0.78 (95% CI 0.65–0.93), 0.79 (95% CI 0.65–0.97), and 0.64 (95% CI 0.51–0.81) to 1.73 (95% CI 1.21–2.47), 1.04 (95% CI 0.69–1.56), and 1.45 (95% CI 0.91–2.31), respectively. In the cycles with elevated progesterone and blastocyst stage transfer, the aORs comparing quartiles 4 with quartile 1 decreased from 0.73 (95% CI 0.62–0.87) and 0.74 (95% CI 0.63–0.87) to 0.58 (95% CI 0.40–0.84) and 0.42 (95% CI 0.25–0.73) than those in the cycles without. However, only blastocyst transfer showed a significant interaction with transfer depth (p = 0.043). Our data suggested that endometrial thickness and blastocyst transfer significantly affect the association between embryo transfer depth and clinical pregnancy.

## Introduction

Embryo transfer is the final and important step toward achieving pregnancy in an in vitro fertilization (IVF) cycle. Even when good quality embryos were created, and a satisfying endometrium was prepared, poor transfer technique may hinder the embryo implantation^1^. Although there is no real consensus on the optimal embryo transfer practice, several factors concerning transfer technique have been associated with IVF outcomes^2,3^.

Among the technical aspects of the transfer procedure that have been studied, the site of embryo transfer has been associated with implantation^3^. However, the evidence available remained conflicting. In the early practice of embryo transfer, the tip of the catheter has been empirically placed 5–10 mm from the uterine fundus^2,4–6^, while other researchers argued that transferring the embryo closer to the cervix^7,8^ or no influence of the depth of the embryo transfer^9^. More recently, Coroleu et al. proposed an optimal positioning of the catheter at 15 to 20 mm from the fundus for ultrasound-guided transfer by showing that positioning the catheter at 10 mm from the fundus significantly decreased the pregnancy rate in their randomized trial containing 180 consecutive patients^10^. A meta-analysis published in 2007 stated that there is limited evidence supporting the superiority of lower cavity transfers compared with the traditional high cavity^11^. Since then, however, negative^12^, positive^13^, or no association^14,15^ between the transfer depth and IVF outcome was still observed by different researchers. By showing that embryo transfer depth was similar between the cycles which led to a pregnancy and those did not, Kovacs et al. argued that transfer depth does not affect implantation and pregnancy rates when the transfer is in the middle or upper third of the uterus^14^. In a prospective study by Rovel et al., higher pregnancy and implantation rates were achieved when the tip was placed between 5 and 15 mm from the fundus compared with > 15 mm distance from the fundus^16^. Finally, an embryo transfer guideline from the American Society for Reproductive Medicine in 2017 concluded that “there is insufficient evidence for more specific recommendations regarding the positioning of the catheter at the time of embryo transfer”^3^.

Given the evidence from the randomized trials remained limited^11^, cohort studies in large populations may contribute to verify the conclusions drawn from the meta-analysis. However, one factor that limited some of the studies examining the role of transfer depth is estimating the effect on pregnancy through simple bivariate analyses, without controlling for confounding effects by other important parameters^12,13^, while several other studies controlled for different sets of confounding factors^16–18^. Many of the important parameters identified in the recent studies that have shown to be associated with the chance of pregnancy, such as endometrial thickness^19^, quality of embryos transferred^18^, number of oocytes retrieved^20^, and progesterone elevation before transfer^21^, were not included in the analyses in many previous studies, even though the multivariate analyses were used. Moreover, some of these confounders, such as progesterone levels^22^, endometrial thickness^23^, and the day of transfer^24^, are not only predictors of implantation but may also affect the frequency and the direction of uterine contraction. Uterine contractions may dislodge embryos and dictate where the embryo will eventually implant following transfer. The association between embryo transfer depth and implantation might be smaller or larger with different degrees of uterine contraction. For instance, in patients with a thin endometrium, Rombauts et al. propose an increased chance of tubal embryo migration^23^. In these patients, embryos transferred near the fundus might move toward the tubal side, distorting the association between transfer depth and implantation.

Theoretically, the significance of the embryo transfer depth is based on its influence on the optimal implantation site. We hypothesize that heterogeneity in important uterine factors may contribute to the lack of consensus in previous studies not only as sources of confounding but also as effect modifiers. Using the distance between the fundal endometrial surface and the air bubbles as a marker of embryo transfer depth, the aim of the study is to explore whether the factors that correlated to uterine contraction modified the effect of transfer depth during embryo transfer.

## Materials and methods

### Study subjects

The retrospective analysis was performed on patients who underwent IVF/ intracytoplasmic sperm injection (ICSI) treatment and fresh embryo transfer in the affiliated Chenggong Hospital of Xiamen University between January 2013 and December 2015. Institutional Review Board approval for this retrospective study was obtained from the Ethical Committee of the Medical College Xiamen University (2018–023). The informed consent was waived by the ethics comment because the research was based on non-identifiable records. All research was performed in accordance with relevant guidelines/regulations.

Only patients undergoing conventional ovarian stimulation (agonist or antagonist) were reviewed. Patients on mild stimulation cycles, natural cycles, and luteal phase stimulation cycles were excluded from the study (n = 177). Forty-eight cases of transfer lacked the record of embryo transfer depth and thus were excluded from the study. We also excluded the patients identified as difficult-to-transfer (n = 100) and the patients who had bacterial infections after the transfer (n = 3). In any of the cases that were examined, there was never a case of blood in the catheter. The details of patient inclusion are shown in supplementary Fig. 1.

### Stimulation protocols and laboratory procedures

In all stimulation cycles, patients received 2–3 ampoules (75–225 IU) of gonadotropin per day during the gonadotropin stimulation. The initial and ongoing dosage was adjusted according to the patient's age, antral follicle count (AFC), body mass index (BMI), and follicular growth response. Recombinant follicle-stimulating hormone (FSH) (Gonal-F; Merck-Serono, Switzerland) or domestic urinary HMG (HMG; Lizhu, China) was used for the gonadotropin stimulation. During the treatment, the ovarian response was monitored by transvaginal ultrasound measurements of follicular growth and serum E_2_ level every 1–3 days. Gonadotropin stimulation continued until ultrasonography revealed at least one follicle measuring ≥ 18 mm in mean diameter. 5000–10000 IU human chorionic gonadotropin (hCG; Lizhu, China) was injected intramuscularly. Endometrial thickness and ultrasonic pattern of the endometrium (Pattern A: a triple-line pattern consisting of a central hyperechoic line surrounded by two hypoechoic layers, pattern B: an intermediate isoechogenic pattern with the same reflectivity as the surrounding myometrium and a poorly defined central echogenic line, and pattern C: homogenous, hyperechogenic endometrium) were also evaluated on the day^25^. The oocyte retrieval was scheduled for 34 to 36 h after hCG administration and carried out under transvaginal ultrasound guidance.

Oocytes were inseminated using either conventional IVF or ICSI. The pronuclei were identified 17 to 18 h later. On day 3, the embryos were assigned quality grades, and the embryos were evaluated according to the number and size of the cells and the degree of fragmentation. For patients receiving blastocyst transfer, the Gardner scale^26^ was used to evaluate the embryo quality. Top-quality embryos for transfer were defined as the following: the embryos with less than 10% fragment and on-time cell size on day3 and good inner cell mass and trophectoderm on day 5.

### Embryo transfer

Fresh embryo transfers were performed on either day 3 or day 5. The patients decided on the day of the embryo transfer with clinical consultation. The number of embryos transferred ranged from 1 to 3 according to the national regulations^27^. Transferring three embryos was only considered in women with advanced age or repeated failure, and no patients had more than two blastocysts transferred.

All transfers were performed in the same room by seven experienced clinicians. Patients undergoing transfer received a mock transfer the day before embryo transfer was performed. All patients were placed in the lithotomy position during the transfer procedure, and the cervix was exposed using a bivalve speculum. The external os was cleaned using a physiologic serum, and the cervical mucus was removed with a cotton swab.

The outer catheter of the Cook catheter (K-JETS-7019-SIVF, Cook, IN, USA) was inserted under the guidance of abdominal ultrasonography. Embryos were loaded to the inner catheter by the ‘three-drop technique’^28^. The drop of medium containing the embryos was separated from a preceding and a following drop of the medium by a bubble of air, and the volume of the air bubble and droplet did not exceed 10 μL.

The embryos were injected with the medium and air bubbles into the uterine cavity at low speed under ultrasonic guidance. The position of injection was addressed to the thickest part of the endometrium as possible^29^. The bubble generated following transfer was visualized under ultrasonography and the distance from the position of the bubble to the fundal myometrium–endometrial interface was used as a marker of the embryo position (embryo transfer depth). The catheter was then gently removed and examined under a stereomicroscope to ensure that all embryos had been transferred. Following the transfer, patients remained in bed for 30 min.

The luteal phase support was sustained with natural progesterone in oil (progesterone; XianJu, China), 60 mg i.m. daily from the oocyte retrieval day. A pregnancy test (serum β-hCG determination) was done 14 days after embryo transfer. Clinical pregnancy was defined as the presence of one or more gestational sacs detected on an ultrasound scan performed 4 weeks after embryo transfer. If no evidence of an intrauterine gestational sac was detected following β-hCG elevation, ectopic pregnancy was confirmed with surgical treatment.

### Statistical analysis

For data analyses, the transfer depth was grouped in all transfer cycles into quartiles. In order to test the effect of extreme values, 10% percentile and 90% of the distance were also used as categorization criteria in multivariate analyses.

For continuous variables, the Q-Q plots were used to evaluate the normality of distribution graphically. The distribution was considered normal when the plot was close to a straight diagonal line. The One-way analysis of variance (ANOVA) for normally-distributed data and the Kruskal Wallis test for non-normally distributed data was used for analyses, respectively. Categorical variables were presented as proportions and percentages of the total. Dichotomous variables were analyzed by chi-square test or Fisher's exact test, as appropriate. When the test was significant (P < 0.05), Bonferroni correction was used for multiple comparisons based on the t-test, Wilcoxon, or chi-square test.

To perform multivariate analyses, the generalized estimating equations (GEE) model was used because one patient may receive multiple transfers in the study. Multivariate analyses were performed to evaluate the association between embryo transfer depth and the probability of clinical pregnancy, with adjustment for important confounding factors. The transfer depth was evaluated either as a categorized value aforementioned or a continuous value (per millimeter increased) in the multivariate analyses. Covariates were selected based on their clinical importance. The model included patient characteristics known to be important for counseling IVF outcomes, such as age, BMI, AFC, previous live birth or pregnancy, duration of infertility, and etiologies of infertility^30^. Stimulation characteristics including stimulation dose, gonadotropin-releasing hormone (GnRH) analogues used^31^, the number of oocytes^11^, endometrial thickness and pattern^25^, and progesterone elevation on the day of triggering^21^ were also selected because they are known to influence the outcomes. Finally, the model was also controlled for other factors that may affect the outcome of embryo transfer, including the development stage of transferred embryos, the presence of at least one good-quality embryo transferred, and different clinicians that performed the embryo transfer.

To explore whether the covariates that correlated to uterine contraction modified the effect of embryo transfer depth, the interaction terms were introduced in the model. The interactions between embryo transfer depth and Blastocyst transfer^24^, progesterone elevation^22^, and endometrial thickness^23^ were studied based on previous knowledge. To facilitate the analysis, the endometrial thickness on the day of hCG was categorized into thin (< 8 mm), normal (8–11 mm), and thick (> 11 mm,) categories. The median values in each transfer depth category was included as a continuous variable to test the overall linear trend across quartiles (p for trend).

All calculations were performed with SPSS (version 19; IBM). In all analyses, P < 0.05 was considered significant, except that the Bonferroni-corrected P-value (P < 0.0125) was used in multiple comparisons.

## Results

Seven thousand eight hundred forty-nine fresh transfer cycles from 6942 patients were included in the present study. The mean age of the patients was 31.44 ± 4.38 years. The transfer depth ranged from 4–25 mm. The 10%, 25%, 50%, 75% and 90% percentile of the distance was 7, 9, 11, 14, 17 mmrespectively. Using quartiles as cut-off values, the cycles were divided into four groups (quartile1-4). In 1735 cycles (22.1%), the embryo transfer depth was ≤ 9 mm (quartile 1). In 2557 cycles (32.6%), the embryo transfer depth was 9.1–11 mm (quartile 2). In 1933 cycles (24.6%), the embryo transfer depth was 11.1–14 mm (quartile 3). And finally, in 1624 cycles (20.7%), the embryo transfer depth was > 14 mm (quartile 4). The mean ± SD of each quartile was 6.754 ± 1.27 mm, 10.01 ± 0.80 mm, 12.83 ± 0.79 mm, and 17.61 ± 2.87 mm, respectively.

The baseline characteristics of patients receiving transfer are summarized in Table 1. The overall baseline characteristics were similar between groups. However, the patients in quartile 2 had a longer duration of infertility, whereas the patients in quartile 4 had fewer previous attempts of transfer, a lower proportion of polycystic ovarian syndrome (PCOS), lower basal luteinizing hormone (LH), and more AFC than the other three groups, as demonstrated by multiple comparisons. In addition, significant heterogeneity in basal FSH levels was noted among groups, but the absolute differences were rather small.

**Table 1:** Baseline characteristics and ovarian stimulation parameters.

Variables	Transfer depth quartiles				P
	Quartile 1 (≤ 9 mm, n = 1735)	Quartile 2 (9.1–11 mm, n = 2557)	Quartile 3 (11.1–13.9 mm, n = 1933)	Quartile 4 (≥ 14 mm, n = 1624)	
**Female`s age, year**					0.400*
Mean(SD)	31.29 ± 4.51	31.45 ± 4.35	31.51 ± 4.41	31.50 ± 4.23	
Median[interquartile range]	31 [28,34]	31 [28,35]	31 [28,34]	31 [28,34]	
**Male`s age, year**					0.279*
Mean(SD)	33.28 ± 5.11	33.45 ± 5.04	33.60 ± 5.09	33.40 ± 4.85	
Median[interquartile range]	33 [28,37]	33 [30,37]	33 [28,37]	33 [28,37]	
**Duration of infertility, year**					0.0357^†^
Mean(SD)	4.61 ± 3.33	4.74 ± 3.41	4.44 ± 3.22	4.65 ± 3.11	
Median[interquartile range]	4.00 [2.00,6.00]	4.00 [2.00,6.00]	4.00 [2.00,6.00]	4.00 [2.00,6.00]	
Primary infertility (%)	804 (46.3)	1236 (48.3)	917 (47.4)	813 (50.1)	0.169
**Previous attempt of ET**					0.0081
0	1345 (77.5%)^d^	2018 (78.9%)^d^	1536 (79.5%)^d^	1354 (83.4%)^abc^	
1	198 (11.4%)	277 (10.8%)	200 (10.3%)	131 (8.1%)	
2	127 (7.3%)	167 (6.5%)	117 (6.1%)	87 (5.4%)	
≧3	65 (3.7%)	95 (3.7%)	80 (4.1%)	52 (3.2%)	
BMI, kg/cm^2^	21.08 ± 2.78	21.17 ± 2.66	21.08 ± 2.92	21.19 ± 2.70	0.461
18.5–24.9	1180 (68.0%)	1753 (68.6%)	1288 (66.6%)	1085 (66.8%)	0.636
< 18.5	330 (19.0%)	464 (18.1%)	370 (19.1%)	299 (18.4%)	
> 24.9	225 (13.0%)	340 (13.3%)	275 (14.2%)	240 (14.8%)	
PCOS (%)	115 (6.6)^d^	159 (6.2)	101 (5.2)	75 (4.6)^a^	0.039
Endometriosis (%)	203 (11.7)	285 (11.1)	237 (12.3)	216 (13.3)	0.200
Hydrosalpinix (%)	75 (4.3)	84 (3.3)	80 (4.1)	46 (2.8)	0.054
Male infertility(%)	222 (12.8)	379 (14.8)	279 (14.4)	253 (15.6)	0.121
**Basal FSH, IU/l**					< 0.001^†^
Mean(SD)	7.51 ± 2.42	7.66 ± 2.60	7.63 ± 2.45	7.42 ± 2.88	
Median[interquartile range]	7.09 [6.01,8.46]	7.28 [6.15,8.71]^d^	7.20 [6.10,8.56]^d^	7.03 [5.99,8.31]^bc^	
**Basal LH IU/l**					0.0111^†^
Mean(SD)	4.92 ± 3.06	4.72 ± 2.85	4.73 ± 2.74	4.59 ± 2.57	
Median[interquartile range]	4.29 [3.18,5.74]^bc^	4.10 [3.10,5.56]^ad^	4.22 [3.18,5.56]^ad^	4.13 [3.03,5.43]^bc^	
**Basal PRL, ng/ml**					0.509^†^
Mean(SD)	15.50 ± 9.79	15.01 ± 9.47	15.52 ± 12.5	15.47 ± 9.52	
Median[interquartile range]	13.9 [9.97,19.0]	13.7 [9.70,18.5]	13.7 [9.85,19.0]	13.9 [9.96,18.9]	
**Basal E**_**2**_,** pg/ml**					0.656^†^
Mean(SD)	45.16 ± 34.7	43.45 ± 25.5	43.74 ± 24.4	43.41 ± 23.8	
Median[interquartile range]	39.0 [28.0,54.0]	40.0 [29.0,53.0]	39.0 [28.8,53.0]	39.0 [28.0,53.0]	
**Basal T, ng/ml**					0.836^†^
Mean(SD)	0.44 ± 1.20	0.53 ± 2.41	0.54 ± 2.48	0.54 ± 2.99	
Median[interquartile range]	0.33 [0.22,0.45]	0.320 [0.220,0.440]	0.330 [0.220,0.450]	0.325 [0.230,0.440]	
**Basal P, ng/ml**					0.784^†^
Mean(SD)	0.85 ± 1.73	0.77 ± 1.07	0.79 ± 1.51	0.83 ± 1.68	
Median[interquartile range]	0.61 [0.360,0.940]	0.610 [0.350,0.910]	0.600 [0.360,0.910]	0.590 [0.350,0.930]	
**AFC**					< 0.001^†^
Mean(SD)	7.91 ± 4.38	7.83 ± 4.21	7.80 ± 4.19	8.19 ± 4.13	
Median[interquartile range]	7.00 [5.00,10.0]^cd^	7.00 [5.00,10.0]^cd^	8.00 [5.00,10.0]^ab^	8.00 [5.00,11.0]^ab^	

Data were presented as mean ± SD and median [interquartile range] for continuous variables, and n (percentage) for categorical variables.

^†^Indicates the data were non-normally distributed and analyzed using Kruskal Wallis test.

*Indicates the data were normally distributed and analyzed using one way ANOVA.

^a^Indicates that the group differs from quartile 1 at p value of 0.0125;

^b^Indicates that the group differs from quartile 2 at p value of 0.0125;

^c^Indicates that the group differs from quartile 3 at p value of 0.0125;

^d^Indicates that the group differs from quartile 4 at p value of 0.0125.

ET, embryo transfer; BMI, body mass index; PCOS, polycystic ovarian syndrome; FSH, follicle-stimulating hormone; LH, luteinizing hormone.; PRL, prolactin; E_2_, estradiol; T, testosterone; P, progesterone; AFC, antral follicle count.

Table 2 presents the ovarian stimulation characteristics, and IVF outcomes in the cycles studied. Besides the transfer depth, significant differences were also noted in GnRH analogues, E_2_ level on the day of hCG, and endometrial thickness and endometrial type on the day of hCG among groups. But the starting and total dose, the oocytes yielded, and the number and quality of embryos transferred were comparable among groups. Bivariate analysis revealed that clinical pregnancy rates and ectopic pregnancy rates were similar across groups.

**Table 2:** Outcome of ovarian stimulation, fertilization and embryo transfer.

Variables	Transfer depth quartiles				P
	Quartile 1 (≤ 9 mm,n = 1735)	Quartile 2 (9.1–11 mm, n = 2557)	Quartile 3 (11.1–13.9 mm, n = 1933)	Quartile 4 (≥ 14 mm, n = 1624)	
Antagonist/agonist (%)	349/1386 (20.1/79.9)^d^	518/2039 (20.3/79.7)^d^	350/1583 (18.1/81.9)	232/1392 (14.3/85.7)^ab^	< 0.001
**Starting dose of stimulation, IU**					0.706^†^
Mean(SD)	208.93 ± 33.60	208.70 ± 34.33	208.89 ± 33.63	208.79 ± 32.32	
Median[interquartile range]	225 [188,225]	225 [188,225]	225 [188,225]	225 [188,225]	
**Total dose of gonadotropin, IU**					0.119*
Mean(SD)	2324.32 ± 624.65	2320.07 ± 621.09	2301.97 ± 609.31	2351.75 ± 604.94	
Median[interquartile range]	2250[1838,2700]	2250[1875,2700]	2250[1800,2700]	2288[1950,2700]	
**E**_**2**_** level on the day of hCG, pg/ml**					0.014*
Mean(SD)	2832.97 ± 1568.52	2719.56 ± 1576.04^d^	2756.70 ± 1578.52	2864.02 ± 1609.90^b^	
Median[interquartile range]	2561[1555,4072]	2457[1408,3935.5]	2500[1457,4034]	2634[1559,4063.75]	
Progesterone elevation, ng/ml	311/1735 (17.9)	432/2557 (16.9)	306/1933 (15.8)	283/1624 (17.4)	0.368
Endometrial thickness, mm	10.02 ± 3.13^a^	10.49 ± 3.18^b^	10.76 ± 2.34^c^	11.63 ± 2.69^d^	< 0.001
Endometrial pattern (%)*					
A	354 (20.4)^cd^	517 (20.2)^d^	365 (18.9)^ad^	271 (16.7)^abc^	< 0.001
B	1247 (71.9)	1787 (69.9)	1341 (69.4)	1058 (65.1)	
C	134 (7.1)	253 (9.9)	227 (11.7)	295 (18.2)	
Number of oocytes retrieved	10.0 [6.00,14.0]^bd^	9.00 [6.00,14.0]^acd^	10.0 [6.00,14.0]^bd^	10.5 [7.00,15.0]^abc^	< 0.001
ICSI cycle(%)	455 (26.2)	704 (27.5)	536 (27.7)	455 (28)	0.65
Blastocyst transfer cycle(%)	149 (8.6)	215 (8.4)	158 (8.2)	149 (9.2)	0.746
**Number of embryos transferred (%)**					
One	361 (20.8/)	512 (20)	400 (20.7)	318 (19.6)	0.060
two	1305 (75.2)	1940 (75.9)	1452 (75.1)	1267 (78)	
three	69 (4)	105 (4.1)	81 (4.2)	39 (2.4)	
At least one top-quality embryo transferred (%)	407 (23.5)	612 (23.9)	441 (22.8)	428/ (26.4)	0.083
Implantation rate, %	50.0 [0,100]	50.0 [0,100]	50.0 [0,100]	50.0 [0,100]	0.244
Ectopic pregnancy** (%)	10/1104 (0.9)	24/1596 (1.5)	15/1212 (1.2)	10/997 (1)	0.496
Clinical pregnancy (%)	1038 (59.8)	1498 (58.6)	1129 (58.4)	921 (56.7)	0.335
Adjusted OR for clinical pregnancy	Ref	0.90 (0.79–1.02)	0.86 (0.74–0.99)	0.70 (0.60–0.82)	< 0.001***

Data were presented as mean ± SD and median [interquartile range] for continuous variables, and n (percentage) for categorical variables.

^†^Indicates the data were non-normally distributed and analyzed using Kruskal Wallis test.

*Indicates the data were normally distributed and analyzed using one way ANOVA.

^a^Indicates that the group differs from quartile 1 at p value of 0.0125;

^b^Indicates that the group differs from quartile 2 at p value of 0.0125;

^c^Indicates that the group differs from quartile 3 at p value of 0.0125;

^d^Indicates that the group differs from quartile 4 at p value of 0.0125.

*Pattern A: a triple-line pattern consisting of a central hyperechoic line surrounded by two hypoechoic layers, pattern B: an intermediate isoechogenic pattern with the same reflectivity as the surrounding myometrium and a poorly defined central echogenic line, and pattern C: homogenous, hyperechogenic endometrium.

**Ectopic pregnancy rate = ectopic pregnancies /(chemical pregnancies + clinical pregnancies + ectopic pregnancies).

***P for trend, ORs were adjusted for female`s age, duration of infertility, hydrosalpinx, the number of oocytes retrieved, starting dose of stimulation, type of GnRH analogues, the number of embryos transferred, endometrial thickness, endometrial pattern, progesterone elevation, the development stage of transferred embryos, the presence of at least one good-quality embryo transferred and providers of embryo transfer.

When adjusted for the aforementioned confounding factors, multivariate analyses revealed a decrease in clinical pregnancy rates in quartile 3 and quartile 4, with quartile 1 as reference. The adjusted odds ratios (aOR) for clinical pregnancy comparing quartile 3 and quartile 4 with quartile 1 were 0.86 (95% CI 0.74–0.99) and 0.70 (95% CI 0.60–0.82), respectively. (Table 2).

**Figure 1:**
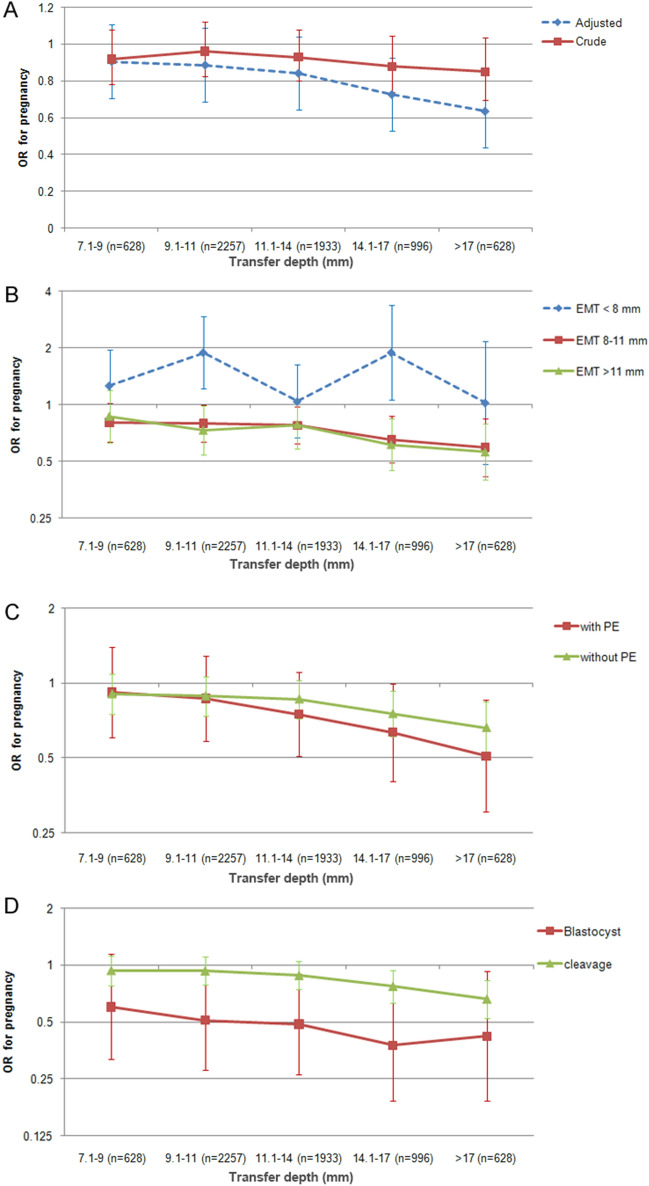
Adjusted ORs (95% CI) for clinical pregnancy adjusted for female`s age, duration of infertility, hydrosalpinx, the number of oocytes retrieved, starting dose of stimulation, type of GnRH analogues, the number of embryos transferred, endometrial thickness, endometrial pattern, progesterone elevation, the development stage of transferred embryos, the presence of at least one good-quality embryo transferred and providers of embryo transfer through different transfer depth levels, using transfer depth ≤ 7 mm (n = 1107) as reference. (**A**) Adjusted and unadjusted ORs for pregnancy. (**B**) Adjusted ORs for pregnancy across endometrial thickness (EMT) categories. (**C**) Adjusted ORs for pregnancy in cycles with and without progesterone elevation (PE) (**D**) Adjusted ORs for pregnancy in cleavage transfer cycles and blastocyst transfer cycles.

To test the effect of extreme values of the transfer depth on clinical pregnancy and illustrate the trend of the change in pregnancy rates across the range of distance, we introduced the 10% and 90% percentile of the transfer depth into analyses. In the six-group comparison using multivariate analysis, the aORs for clinical pregnancy of different distances (7.1–9 mm, 9.1–11 mm, 11.1–14 mm, 14.1–17 mm and > 17 mm) in comparison with the distance of ≤ 7 mm was 0.91 (95% CI 0.76–1.08), 0.89 (95% CI 0.75–1.05), 0.84 (95% CI 0.72–0.99), 0.73 (95% CI 0.60–0.88) and 0.64 (95% CI 0.51–0.80) respectively. A trend of decrease in clinical pregnancy with the increase of transfer depth is illustrated in Fig. 1. The P-value for the trend was less than 0.001.

To explore whether the association between embryo transfer depth and pregnancy differs across stratum of potential effect modifiers, the interaction terms of endometrial thickness × embryo transfer depth, blastocyst transfer × embryo transfer depth, progesterone elevation × embryo transfer, and transfer provider × embryo transfer depth were introduced into the model. When the endometrial thickness on the day of hCG was categorized into thin (< 8 mm, n = 913), normal (8–11 mm, n = 3760), and thick (> 11 mm, n = 3176) categories, we found a significant interaction (P = 0.01). The size of association comparing quartile 2, quartile 3, and quartile 4 with quartile 1 in the thin group was 1.96 (95% CI 1.33–2.90), 1.20 (95% CI 0.78–1.87), and 1.98 (95% CI 1.20–3.26) times than those in the normal group, suggesting an effect modification of thin endometrium. In comparison with that in the cycles with a normal endometrial thickness (8–11 mm), the aORs comparing quartiles 2 through 4 with quartile 1 in the cycles with an endometrial thickness < 8 mm increased from 0.78 (95% CI 0.65–0.93), 0.79 (95% CI 0.65–0.97), and 0.64 (95% CI 0.51–0.81) to 1.73 (95%: 1.21–2.47), 1.04 (95%: 0.69–1.56), and 1.45 (95%: 0.91–2.31), respectively. In contrast, the size of association comparing quartile 2, quartile 3, and quartile 4 with quartile 1 in the thick group were 1.07 (95% CI: 0.79–1.45), 1.09 (95% CI: 0.80–1.50), and 1.03 (95% CI: 0.74–1.43) times than those in the normal thickness group (Table 3).

**Table 3:** Association between embryo transfer depth and pregnancy rate in subgroups and interaction analyses.

	Adjusted ORs (95% CI) in subgroups			change of association across subgroups	
	Normal	Thin	Thick	Thin vs normal	Thick vs normal
	Endometrial thickness				
Quartile 1 (≤ 9 mm, n = 1735)	Ref	Ref	Ref		
Quartile 2 (9.1–11 mm, n = 2557)	0.78 (0.65–0.93)	1.73 (1.21–2.47)	0.84 (0.66–1.06)	1.96 (1.33–2.90)	1.07 (0.79–1.45)
Quartile 3 (11.1–13.9 mm, n = 1933)	0.79 (0.65–0.97)	1.04 (0.69–1.56)	0.85 (0.66–1.09)	1.20 (0.78–1.87)	1.09 (0.80–1.50)
Quartile 4 (≥ 14 mm, n = 1624)	0.64 (0.51–0.81)	1.45 (0.91–2.31)	0.64 (0.50–0.82)	1.98 (1.20–3.26)	1.03 (0.74–1.43)

	Stage of embryo transfer				
	Cleavage	Blastocyst		Blastocyst vs cleavage	
Quartile 1 (≤ 9 mm, n = 1735)	Ref	Ref			
Quartile 2 (9.1–11 mm, n = 2557)	0.94 (0.82–1.08)	0.53 (0.33–0.86)		0.52 (0.32–0.85)	
Quartile 3 (11.1–13.9 mm, n = 1933)	0.90 (0.77–1.04)	0.53 (0.32–0.89)		0.60 (0.36–1.00)	
Quartile 4 (≥ 14 mm, n = 1624)	0.74 (0.63–0.87)	0.42 (0.25–0.73)		0.55 (0.33–0.93)	

	Progesterone elevation				
	No	Yes		With progesterone elevation vs without	
Quartile 1 (≤ 9 mm, n = 1735)	Ref	Ref			
Quartile 2 (9.1–11 mm, n = 2557)	0.9 (0.78–1.04)	0.81 (0.58–1.12)		0.86 (0.68–1.07)	
Quartile 3 (11.1–13.9 mm, n = 1933)	0.88 (0.75–1.03)	0.72 (0.50–1.02)		0.79 (0.60–1.03)	
Quartile 4 (≥ 14 mm, n = 1624)	0.73 (0.62–0.87)	0.58 (0.40–0.84)		0.77 (0.58–1.01)	

All models were adjusted for female`s age, duration of infertility, hydrosalpinx, the number of oocytes retrieved, starting dose of stimulation, type of GnRH analogues, the number of embryos transferred, endometrial thickness, endometrial pattern, progesterone elevation, the development stage of transferred embryos, the presence of at least one good-quality embryo transferred and providers of embryo transfer.

On the other hand, both progesterone elevation and blastocyst transfer decreased the ORs. In the cycles with elevated progesterone and blastocyst stage transfer, the aORs comparing quartile 4 with quartile 1 decreased from 0.73 (95% CI 0.62–0.87) and 0.74 (95% CI 0.63–0.87) to 0.58 (95% CI 0.40–0.84) and 0.42 (95% CI 0.25–0.73) than those in the cycles without. Interaction analyses showed that the size of association comparing quartiles 2 through 4 with quartile 1 was 0.52 (95%: 0.32–0.85), 0.60 (95%: 0.36–1.00), and 0.55 (95%: 0.33–0.93) times comparing blastocyst transfer with cleavage stage transfer, indicating a significant effect of interaction (P for interaction term was 0.045). On the other hand, the size of association in cycles with elevated progesterone was 0.86 (95%: 0.68–1.07), 0.79 (95%: 0.60–1.03), and 0.77 (95%: 0.58–1.01) times comparing cycles with progesterone elevation with those without (P for interaction term was 0.43). Interaction (P for interaction term was 0.25) was not detected between embryo transfer providers and transfer depth (Supplemental Table 2).

When the transfer depth was treated as a continuous value, the aOR for clinical pregnancy per millimeter increased was 0.97 (95% CI: 0.96–0.99) in multivariate analyses (Supplemental Table 1). The aORs for clinical pregnancy of other covariates, which were included in multivariate analyses, are also presented in Supplementary Table 1. The association between continuous transfer depth and pregnancy also differs between the cycles with a thin endometrium and the cycles with a normal endometrium, as well as between the cycles with blastocyst transfer and the cycles with cleavage stage transfer, but not between the cycles with progesterone elevation and without (Supplemental Fig. 2). When the interaction terms were included in the models, the size of the association between transfer depth and clinical pregnancy in the thin group was 1.05 (95% CI 1–1.09) times than those in the normal group, indicating a significant interaction (P = 0.043). On the other hand, the size of the association between transfer depth and clinical pregnancy in the thick group was 1 (95% CI 0.97–1.03) times than those in the normal thickness group (P = 0.929). In cycles with blastocyst stage transfer, the size of association was 0.97 (95% CI 0.93–0.99) times than the cycles without (P = 0.047). No significant interaction was detected between transfer depth and progesterone elevation (P = 0.73).

## Discussion

In the present study, we demonstrated a negative association between the embryo transfer depth and pregnancy rate in a multivariate analysis containing 7849 fresh embryo transfer cycles. Moreover, our data suggested that the uterine factors that have been associated with changes in uterine contraction, including thin endometrium and blastocyst stage transfer might modify the association between embryo transfer depth and pregnancy.

Efforts to find an ideal embryo transfer depth during the transfer procedure have been challenged by findings suggesting that embryos might undergo significant migration following replacement^32–35^. Saravelos et al. suggested that uterine factors such as uterine contractility may dictate where the embryo will eventually implant following transfer^35^. Several studies demonstrated that embryos might undergo significant migration following replacement^32–35^. However, the movement of embryos following the transfer was not random and most of the embryo flashes (the ultrasonic marker of embryo position) underwent migration towards the fundus or remained static 60 min following transfer^34^. The pregnancy rates among patients with embryo flashes located < 15 mm from the fundus at 60 min post-transfer were still significantly higher than those with embryo flashes located > 15 mm from the fundus^34^. Therefore, it is suspected that the combination of uterine contraction and embryo transfer technique may determine the final location of implantation and thus affect the chance of pregnancy.

Rombauts et al. showed that a thinner endometrium is associated with increased ectopic pregnancy risk, whereas increased endometrial thickness is associated with higher placenta praevia risk^23,36^, proposing that increased endometrial thickness is considered as a marker for increased fundus-to-cervix uterine peristalsis^23^. It hinted that the directionality of embryo dislodging after transfer might differ in patients with different endometrial thicknesses: patients with a thin endometrium are more likely to undergo a tubal embryo migration, resulting in an increased ectopic pregnancy rate while patients with a thicker endometrium might expel the embryos to the cervix direction. Our study showed that the association between transfer depth and pregnancy rates might differ across endometrial thickness categories. The adjusted ORs were significantly increased in cycles with lower endometrial thickness, suggesting a detrimental effect on the pregnancy rate of deep fundus transfer in patients with a thin endometrium. The observation may support the hypothesis that the endometrial thickness is associated with the directionality of uterine peristalsis and further affects the embryo migration following transfer.

Known as a relaxant of uterine contraction, progesterone level is another factor that may affect the embryo deposition^22,24^. Fanchin et al. suggested that uterine contraction frequency decreased in the patients with high progesterone levels and was negatively correlated with progesterone concentrations on the day of embryo transfer^22^. Similarly, increased progesterone levels during the luteal phase may also decrease uterine contractility after blastocyst transfer^24^. In such situations, the size of the association between embryo transfer depth and pregnancy was decreased. It is possible that with decreased uterine contraction frequency, the embryos are less likely to migrate away from their initial location and the embryo transfer depth is more likely to reflect the embryo`s position after transfer. The data suggested the importance of considering the patients` uterine environment when evaluating the association between transfer depth and pregnancy rates.

The progesterone levels may also be affected by the luteal phase support if the support is started on the day of retrieval. In comparison with the luteal phase method we used in our study, vaginal administration of luteal phase support may result in high uterine levels of progesterone with low systemic exposure^37^. Whether the type of luteal phase support used before embryo transfer further affects the uterine contraction, the embryo location, or the efficiency of embryo transfer may need further investigation.

Although conflicting with several previous studies^10,12,14,15,17^, the positive association between transfer depth and implantation observed in our study was consistent with the report of Pacchiarotti et al.^13^, and echoed several observations in the early days^4–6^. The lower adjusted pregnancy rates observed in cycles with embryos transferred at a distance > 14 mm from the fundus also partially confirmed the results of Rovei et al., which suggested that a transfer distance above 15 mm compromised the implantation and pregnancy rates^16^. The results were also logically in line with the studies suggesting an embryo position closer to the fundal myometrium–endometrial interface results in a better chance of pregnancy^15,38,39^. More recently, Bayram et al. further confirmed the negative association between the transfer depth and implantation in a cohort of euploid blastocyst transfer cycles^18^.

A significant concern on transferring embryos close to the fundus is that placing the catheter tip near the fundus might transfer the embryos into the tube, possibly leading to ectopic pregnancies^8,40^. In our study, no significant difference in the ectopic pregnancy rates among groups was observed. However, given the total events of ectopic pregnancies were relatively rare, it is immature to draw a firm conclusion in this regard from the present data.

Due to the limitation of retrospective nature, there were a number of residual or unmeasured confounding that might still be present in the present study. Many other parameters during the embryo transfer procedure, such as fundal level of the uterine cavity, length of the uterine cavity, and transfer speed, were not recorded in the study, and thus the interactions between the parameters were unknown. Patients' individual anatomy and providers' preferences also affect the embryo transfer procedure. Although we excluded the difficult-to-transfer patients from the study and controlled for a high number of variables including different clinicians providing the transfer, the potential biases introduced by unknown/unmeasured factors should still be considered.

In summary, we reexamined the effect of embryo transfer depth on pregnancy rates in an IVF population containing 7849 cycles and suggested that factors associated with uterine contraction, including thin endometrium and blastocyst transfer, significantly affect the association between embryo transfer depth and clinical pregnancy. The potential modification effects of uterine-related factors may partially explain the heterogeneity among studies and warrant future studies on individualized embryo transfer. The finding may also suggest a need for an individualized embryo transfer strategy. The embryo location in the uterus following transfer is determined by many parameters, such as uterine orientation, the distance of the catheter tip to the fundus, and injection speed^41^. For instance, a suitable distance of the catheter tip to the fundus (~ 10 mm) may maximize the chance of the embryo to locate at a position near the fundus and a medium injection speed (~ 3μL/min) is more likely to locate the embryo in the static region^41^. Careful adjustments of these parameters during embryo transfer may benefit certain patients, such as patients with a thin endometrium. On the other hand, however, the practices of embryo transfer vary widely and standardization of parameters is still lacking^42^. Future prospective studies are needed to explore the optimal parameter combination in different patients.

## Supplementary Information

Supplementary Information.

## Data Availability

The datasets generated and/or analysed during the current study are not publicly available due to the limitation of the institutional regulation but are available from the corresponding author on reasonable request.
