# Supplementary material for: Uterine factors modify the association between embryo transfer depth and clinical pregnancy
Source: Sci Rep. 2022 Aug 22;12:14269. doi: 10.1038/s41598-022-18636-4 (PMC9395418; doi:10.1038/s41598-022-18636-4)
Supplement: Supplementary file 1 — Supplementary Information. [file 41598_2022_18636_MOESM1_ESM.doc]

Supplementary Information

Uterine factors modify the association between embryo transfer depth and clinical pregnancy

Xiaohua Sun^a^, Jiali Cai^a^, Lanlan Liu ^a^, Haixiao Chen ^a^, Xiaoming Jiang^a^, Jianzhi Ren^a，*^

^a^The Affiliated Chenggong Hospital of Xiamen University, Xiamen, Fujian, 361002, China.

^*^Correspondence address. Tel: + 086-592-6335275; Fax: + 086-592-6335530; E-mail: rjz174@126.com


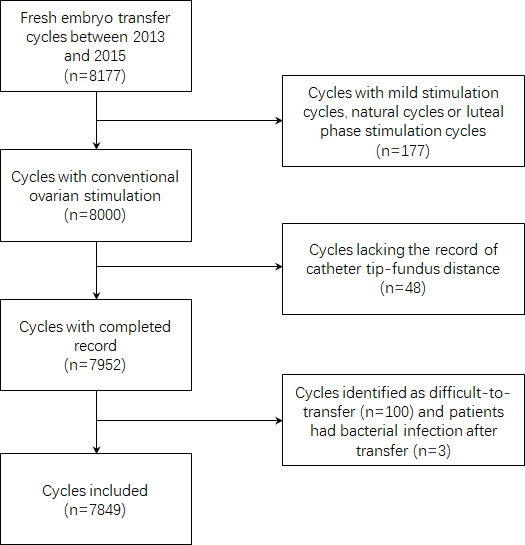


Figure S1 flow chart of patient inclusion.


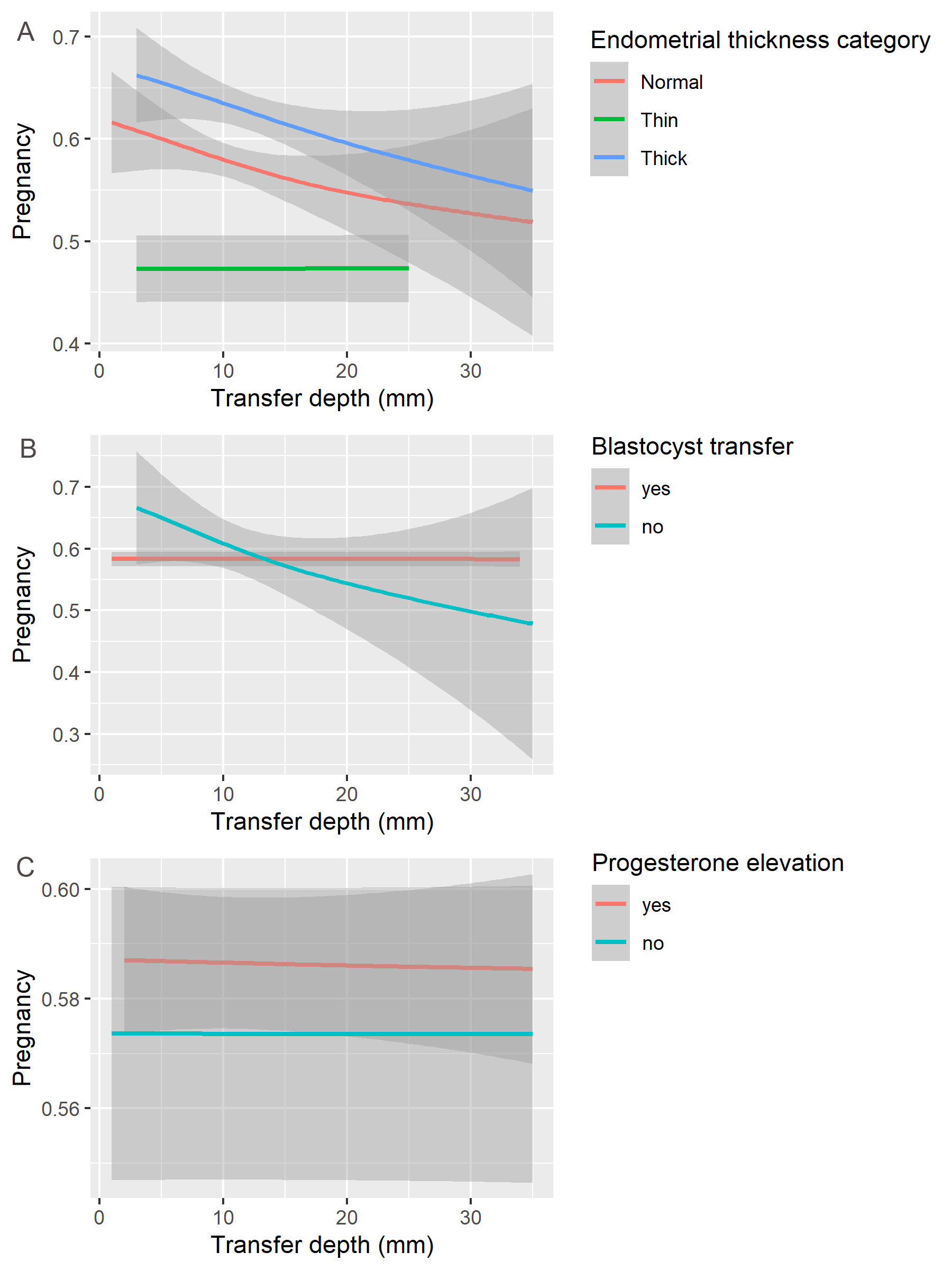


Figure S2. Association between transfer depth and clinical pregnancy, categorized by (A) endometrial thickness, (B) blastocyst transfer, and (C) progesterone elevation. Shade indicates 95% confidence intervals.

Supplemental Table 1 Logistic regression analysis of clinical pregnancy per transfer

| Parameters | Categories | OR (95% CI) |
| --- | --- | --- |
| Female` age | per year increased | 0.97(0.96-0.98) |
| GnRH analogues | Antagonist vs agonist | 0.57(0.49-0.65) |
| Previous ET | Per ET increased | 0.98(0.92-1.04) |
| Type of fertility | Primary vs Secondary | 0.98(0.88-1.09) |
| Hydrosalpinix | with vs without | 0.58(0.45-0.74) |
| PCOS | with vs without | 1.10(0.86-1.39) |
| Endometriosis | with vs without | 0.99(0.86-1.16) |
| Male factor | with vs without | 1.09(0.94-1.26) |
| Duration of infertility | per year increased | 0.97(0.95-0.98) |
| AFC | per AFC increased | 1.00(0.98-1.02) |
| Starting dose of stimulation | per 75 IU increased | 0.75(0.65-0.87) |
| Progesterone elevation | with vs without | 0.82(0.72-0.93) |
| Endometrial thickness | per mm increased | 1.06(1.03-1.08) |
| Endometrial pattern | pattern B vs pattern A | 0.96(0.83-1.07) |
|  | pattern C vs pattern A | 0.79(0.66-0.95) |
| Number of oocytes retrieved | per oocyte increased | 1.02(1.01-1.04) |
| Developmental stage of embryo transferred | blastocyst vs cleavage | 1.95(1.55-2.44) |
| Number of embryo transferred | two vs one | 2.8(2.43-3.30) |
|  | three vs one | 2.84(2.14-3.79) |
| At least one top-quality embryo transferred | yes vs no | 1.37(1.21-1.53) |
| Transfer providers | provider B vs provider A | 0.99(0.83-1.19) |
|  | provider C vs provider A | 1.33(1.12-1.58) |
|  | provider D vs provider A | 0.97(0.80-1.17) |
|  | provider E vs provider A | 1.13(0.96-1.34) |
|  | provider F vs provider A | 1.11(0.91-1.36) |
|  | provider G vs provider A | 0.96(0.73-1.25) |
| BMI categories | <18.5 kg/m^2^ vs 18.5-24.9 kg/m^2^ | 0.85 (0.74-0.99) |
|  | ≥25 kg/m^2^  vs 18.5-24.9 kg/m^2^ | 0.94(0.76-1.17) |
| Transfer depth | per mm increased | 0.97(0.96-0.99) |

Supplemental Table 2 Interaction between transfer providers and the effect of transfer depth on clinical pregnancy rates, derived from a multivariate logistic regression model with live birth as a dependent variable and transfer depth, transfer providers, the interaction term: catheter tip-fundus×transfer providers, age, GnRH analogues, starting dose of stimulation, number of oocytes, endometrial thickness, endometrial pattern, progesterone elevation, number and developmental stage of embryos transferred, presence of top quality embryo, duration of fertility and hydrosalpinix as independent variables)

|  | Ratio of OR for clinical pregnancy | | |
| --- | --- | --- | --- |
|  | Quartile 2 vs Quartile 1 | Quartile 3 vs Quartile 1 | Quartile 4 vs Quartile 1 |
| provider B vs provider A | 0.74(0.46-1.20) | 0.69(0.41-1.16) | 1.03(0.57-1.86) |
| provider C vs provider A | 1.15(0.71-1.85) | 1.25(0.75-2.09) | 1.87(1.05-3.33) |
| provider D vs provider A | 0.67(0.42-1.07) | 0.78(0.46-1.32) | 1.17(0.63-2.15) |
| provider E vs provider A | 0.94(0.61-1.44) | 1.03(0.64-1.65) | 1.16(0.66-2.03) |
| provider F vs provider A | 1.00 (0.61-1.66) | 1.02(0.58-1.79) | 1.19(0.63-2.27) |
| provider G vs provider A | 0.67 (0.35-1.30) | 0.54 (0.25-1.17) | 2.25(0.91-5.60) |
